# Extremes of summer climate trigger thousands of thermokarst landslides in a High Arctic environment

**DOI:** 10.1038/s41467-019-09314-7

**Published:** 2019-04-02

**Authors:** Antoni G. Lewkowicz, Robert G. Way

**Affiliations:** 1 Department of Geography, Environment and Geomatics, University of Ottawa, Ottawa, ON K1N 6N5 Canada; 2 Department of Geography and Planning, Queen’s University, Kingston, ON K7L 3N6 Canada

## Abstract

Retrogressive thaw slumps (RTS) – landslides caused by the melt of ground ice in permafrost – have become more common in the Arctic, but the timing of this recent increase and its links to climate have not been fully established. Here we annually resolve RTS formation and longevity for Banks Island, Canada (70,000 km^2^) using the Google Earth Engine Timelapse dataset. We describe a 60-fold increase in numbers between 1984 and 2015 as more than 4000 RTS were initiated, primarily following four particularly warm summers. Colour change due to increased turbidity occurred in 288 lakes affected by RTS outflows and sediment accumulated in many valley floors. Modelled RTS initiation rates increased by an order of magnitude between 1906–1985 and 2006–2015, and are projected under RCP4.5 to rise to >10,000 decade^−1^ after 2075. These results provide additional evidence that ice-rich continuous permafrost terrain can be highly vulnerable to changing summer climate.

## Abstract (web summary)

Retrogressive thaw slumps (RTS) are landslides caused by melting ground ice in permafrost areas. Based on Google Earth Engine Timelapse data, the authors show an unprecedented 60-fold increase in RTS numbers for Banks Island, Canada, from 1984 to 2015 due to a warming summer climate.

## Introduction

Ground ice melt associated with thawing permafrost (i.e. thermokarst) can profoundly affect arctic landscapes and ecosystems^1–4^. Thermokarst landforms include thawing ice wedge networks^5,6^, degrading peat plateaus^7^ and palsas^8,9^, and on slopes, active layer detachments^10,11^ and retrogressive thaw slumps (RTS)^12^. In most cases, the loss of ground ice associated with the formation of these features leads to surface collapse which is irreversible over time scales of decades to centuries, as well as liberating previously frozen carbon^13^. Several investigations have shown enhanced thermokarst activity in the Arctic associated with climate warming^5,7^ and/or an increase in precipitation^14–16^. In this study we focus on changes to the rate of formation of RTS and the links between summer climate and the initiation of these rapidly evolving and visually striking thermokarst landforms.

**Fig. 1:**
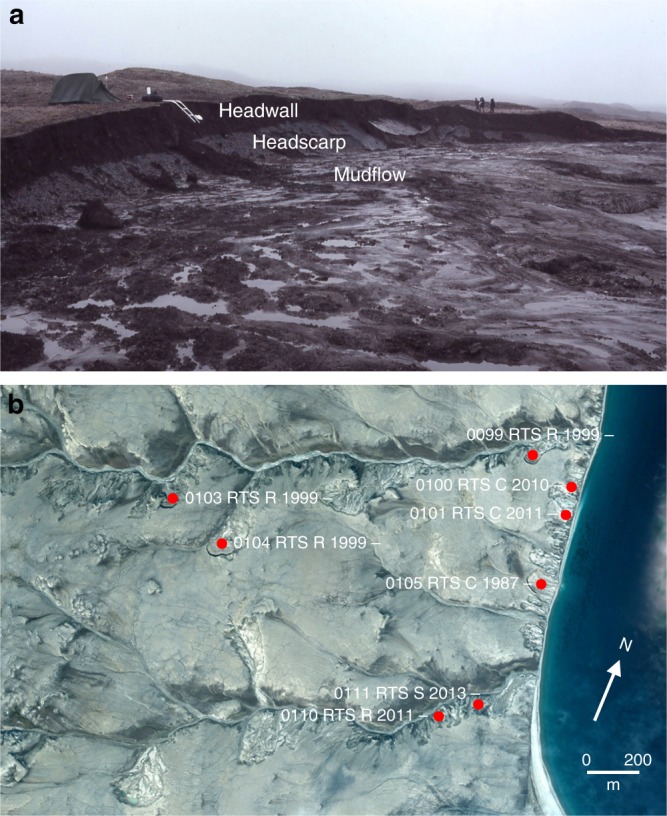
Ground and satellite views of retrogressive thaw slumps. **a** Polycyclic coastal retrogressive thaw slumps in southwest Banks Island (71.717°N, 124.127°W). Headscarp is thawing ice-rich permafrost (averaging 85% ice by volume^18^) while overlying headwall is the former stabilised mudflow comprising the active layer and ice-poor permafrost. Undercutting of the headwall by ablation of the ground ice results in soil collapse that temporarily covers the ice. **b** Quickbird image in Google Earth of part of the coast of eastern Banks Island (centred on 72.18°N 120.19°W) showing those retrogressive thaw slumps identified as active between 1984 and 2015 using the Timelapse dataset. Individual retrogressive thaw slumps are tagged with an identification number, the location of initiation (R-river, C-coast, L-lake or S-slope) and their years of activity. Where the start of thaw slumping post-dates the date of image acquisition (2004) the outline of the landform is not present. The absence of a final date means that a retrogressive thaw slump was still active at the end of the Timelapse period. Note the many unnumbered retrogressive thaw slump scars where thermokarst activity was not observed during the Timelapse period

An RTS comprises a headscarp of thawing ice-rich sediments or massive ice, an overlying headwall composed of the active layer and low ice-content permafrost, and a bowl downslope filled with mud and debris derived from meltwater and soil from the collapse of the under-cut headwall^12^ (Fig. 1a). Once initiated by the exposure of ground ice, RTS enlarge by retrogression at typical rates of 5–15 m yr^−1 ^^3,12,16^ so that directly disturbed areas increase through time. RTS stabilise in autumn as air temperatures drop below 0 °C and melting of the ground ice ceases. They reactivate in summer, providing debris covering the headscarp can flow away, re-exposing the ground ice. A single retrogression of the headscarp, which can continue for as long as 50 years^3^, may result in incomplete thaw of the ice-rich layer of permafrost because the mudflow can preserve ground ice beneath it. This preserved ice may be subsequently exposed, resulting in a polycyclic^14,17–19^ headscarp retrogressing upslope in the floor of a stabilised RTS. Re-exposure can occur as a result of renewed fluvial incision or coastal erosion at the base of the slope, or due to detachment failure associated with deep or rapid thaw within the RTS floor^20,21^. The consequence is that a given site can be repeatedly affected by RTS activity.

RTS are localized terrain disturbances, but when present in high concentrations, they impact stream sediment and solute transport^22,23^, lake water quality^24^, coastal erosion and sedimentation^20,23,25^, and carbon cycling^1,13,26^. A warming climate can affect the energy balance of the headscarp and hence ice ablation and retrogression rates, by altering fluxes of net radiation (e.g. through changes in cloudiness) or sensible heat (e.g. through changes in air temperature or wind speed)^18,27^. More importantly, RTS initiation rates and spatial distribution could be affected by climate change^20,23,28,29^. Inuvialuit (local Inuit) from Banks Island, Canada, reported in 1999–2000 that the numbers of exposures of ground ice and slumps were increasing, and that RTS had become common inland, not just at the coast^30^. Such observations have been challenging to examine in detail, however, due to the episodic nature of RTS initiation and the absence of continuous long-term records.

Here we use the Google Earth Engine Timelapse dataset^31^ (see Methods), covering the period 1984–2016 and accessed through a web-based interface, to generate novel information regarding RTS activity. Our analyses cover the whole of Banks Island where several recent studies have shown locally high concentrations of RTS^15,23,28^, as well as increases in thermokarst activity associated with ice wedge melt^5^. We document the year of feature initiation, the longevity of RTS, the location of initiation in the landscape (Fig. 1b), and the relation of RTS activity to other landscape change over an area of 70,000 km^2^.

## Results

### Change in RTS activity (1984–2015)

**Fig. 2:**
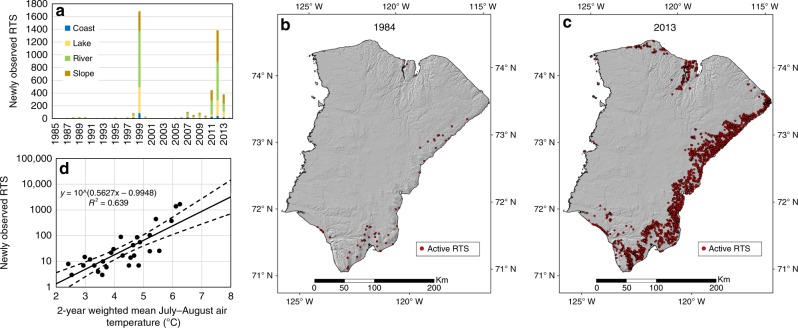
Observations of active retrogressive thaw slumps on Banks Island (1984–2014). **a** Annually resolved numbers of newly observed slumps (1985–2014) classified by location of initiation. Potential errors (not shown) are assessed as −5% to +10% (see Methods). **b** Distribution of 63 retrogressive thaw slumps observed to be active in 1984 at the start of the Timelapse period. **c** Distribution of retrogressive thaw slumps active in 2013 when numbers peaked at 4077. **d** Relationship between 2-year weighted mean July–August air temperature (derived from the Hadley CRU dataset^52,53^) and the number of newly observed retrogressive thaw slumps. Solid line is best-fit and dashed lines are 95% confidence intervals

We observed a remarkable increase in RTS activity (Fig. 2a). Only 63 RTS appeared active on the entire island in 1984 (Fig. 2b). Over the next three decades, more than 4500 RTS were initiated and the number of active RTS increased 60-fold, reaching a maximum of 4077 in 2013 (Fig. 2c). 86% of these RTS were newly observed in the imagery from four of the 32 years: 1999 (1682 RTS), 2011 (445 RTS), 2012 (1385 RTS) and 2013 (379 RTS) (Fig. 2a).

The number of newly observed RTS was clearly linked to summer air temperature. It was predictable using a log-linear relation with a 2-year average of July–August air temperature weighted between the preceding summer (66.7%) and the current year (33.3%) (Fig. 2d). The weighting reflects the possibility that RTS may become visible on Timelapse imagery in the actual year of their initiation or, more frequently, in the following year. The log-linear form of the relationship underlines that it is extreme positive summer air temperatures that are most significant to RTS initiation^14^.

**Fig. 3:**
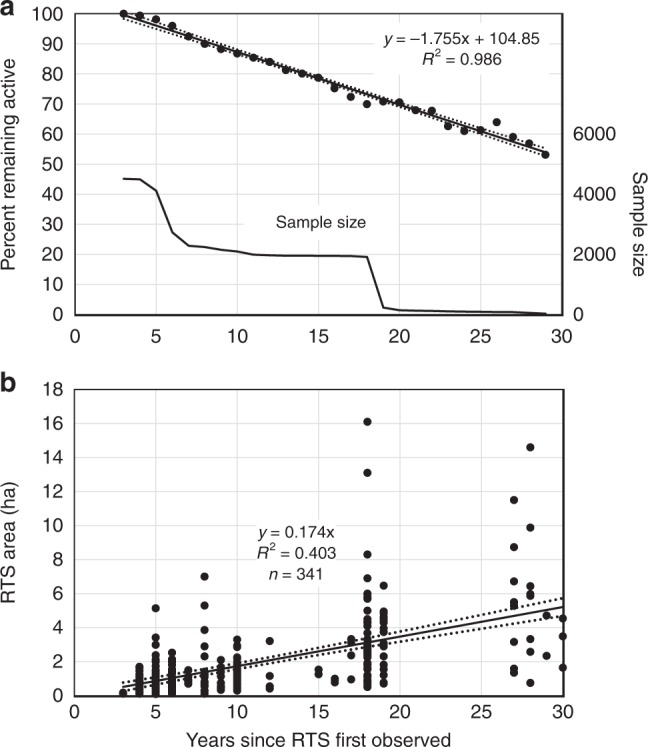
Longevity and growth of retrogressive thaw slumps. **a** Percent of all observed features remaining active for a given duration. Best-fit relation is for retrogressive thaw slumps >2 years old and for sample size >30 (upper solid line). Dotted lines are 95% confidence intervals. Lower solid line is sample size. All retrogressive thaw slumps were active for at least 3 years. **b** Measured area of sampled active retrogressive thaw slumps against age. Best-fit line was constrained through the origin. Dotted lines are 95% confidence intervals

**Fig. 4:**
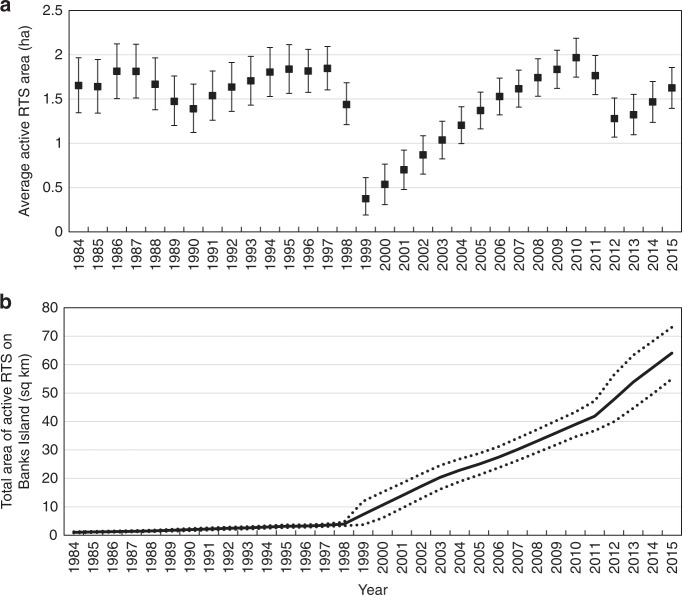
Calculated area of active retrogressive thaw slumps on Banks Island (1984–2015). **a** Average feature area based on population age distribution in each year and the area-age relationship in Fig. 3b. Error bars are 95% confidence intervals. **b** Total area of active retrogressive thaw slumps. Dotted lines are 95% confidence intervals

RTS initiated since 1984 had a 70% probability of remaining active for more than 20 ± 1 years with 50% expected to remain active for longer than 31 ± 1 years, enlarging throughout this period (Fig. 3a). Using a sample of RTS areas (*n* = 341) (Fig. 3b), we calculate that active RTS on Banks Island occupied a total area of 1.0 km^2^ in 1984 but by 2015 this had greatly increased to 64.1 km^2^ (range 55.0–73.2 km^2^) with a rate of annual expansion of 5.1 ± 0.2 km^2^ yr^−1^ (Fig. 4b). The average RTS area was 1.63 ± 0.23 ha in 2015, but this varied through the years from a minimum of 0.37 (0.19–0.61) ha in 1999 to a maximum of 1.97 (1.75–2.19) ha in 2010 (Fig. 4a). These variations reflect the formation of numerous small RTS during major initiation events and their subsequent enlargement through time. The largest single RTS had an area of 16 ha in 2015, which is smaller than mega-slumps farther south^32^, but the latter were surpassed in area by several laterally conjoined RTS which occupied 125 ha and extended along a lakeshore for 2.5 km (Supplementary Table 1, Supplementary Video 1).

Assuming an average elevation loss associated with headscarp retrogression of 1–2 m on Banks Island^33^, the ice volume lost due to RTS activity from 1985–2015 is calculated to be 63.1–126.2 × 10^6^ m^3^ (range 54.0–146.4 × 10^6^ m^3^). This is equivalent to a mass loss of 0.058–0.116 Gt (range 0.050–0.134 Gt) over three decades.

### Spatial distribution

**Fig. 5:**
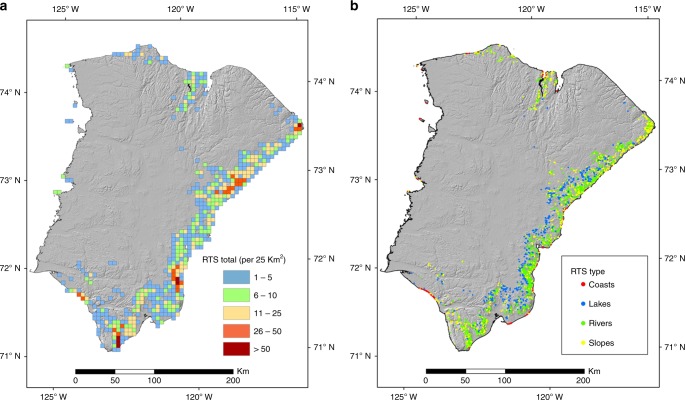
Distribution of all active retrogressive thaw slumps (1984–2015). **a** Spatial concentration (25 km^2^ grid cells). **b** Distribution by locus of initiation

RTS activity was spatially concentrated, with most occurring in a 25–50 km wide band adjoining the eastern, southern and northern coasts of the island (Fig. 2c), a distribution associated with the maximum extent of Late Wisconsinan glacial ice cover^15,34^. The area with active RTS comprised 17.3% of the 25 km^2^ grid cells covering the island (Fig. 5a). A small number of RTS were present on the west coast, including on offshore islands, and in interior areas along the track of ice that retreated southwards in the late Wisconsinan^34^. Maximum densities by number or by area affected were higher than previously reported^23,28^ reaching 18 RTS in a 1 km^2^ grid cell and 88 RTS in a 25 km^2^ cell (Supplementary Video 2). Using the average RTS area in 2015, these correspond respectively to disturbance totalling 29 ha km^−2^ and 141 ha in 25 km^2^. Although active RTS cover only about 0.1% of the entire island, this extent is several orders of magnitude greater than in recently studied transects elsewhere in the Arctic^35^. This spatial clustering, linked to ground ice content and Quaternary history, underlines the heterogeneity of permafrost landscape vulnerability to climate warming^1,15^.

**Fig. 6:**
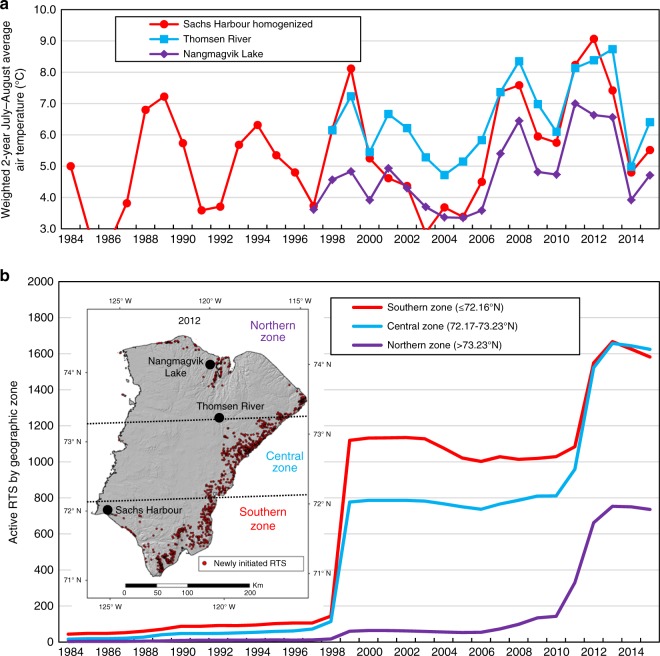
Spatial response of retrogressive thaw slump initiation to differential warming. **a** Weighted 2-year July–August average air temperature at three official climate stations on Banks Island: Sachs Harbour (1984–2015), Thomsen River (1998–2015) and Nangmagvik Lake (1997–2015). **b** Total active retrogressive thaw slumps in southern (≤72.16°N—red line), central (72.17–73.23°N—blue line) and northern (>73.23°N—purple line) zones. Inset map shows the boundaries of the three zones, the location of the three climate stations, and retrogressive thaw slumps first observed in 2012

Most RTS were active in the southern and central parts of Banks Island prior to 2007 (Fig. 6). In the last nine years of the record, however, >500 RTS were initiated in the northern third of the island. This significant areal expansion coincided with increases in July–August air temperature measured in this part of the island (Fig. 6a).

RTS were initiated most frequently next to rivers (45%), and less often on slopes (27%), around lakes (23%) and at the coast (5%) (Fig. 5b; Supplementary Videos 3–6). As observed by Inuvialuit^30^, coastal RTS became proportionally less important during the record, declining from 24% of the overall RTS population in 1984 to 6% in 2015 (Fig. 2a). Notwithstanding relative change by locus of initiation, each of the four types of RTS increased by at least 20 times in absolute numbers.

The finding that all types of RTS increased in the major initiation years (Fig. 2a) is unexpected. There is no obvious reason why processes as diverse as fluvial, lacustrine and coastal erosion or long-term talik development adjacent to lakes^19^, all previously linked to the initial exposure of ground ice^3,12^, should initiate large numbers of RTS in the same year. Our results, therefore, suggest an alternate mechanism. In a warm summer, as with active layer detachment formation, thaw consolidation at the base of the active layer or in the thawing transient layer leads to high porewater pressures, a reduction in effective shear strength, and slope failure where the factor of safety falls below unity^10,36,37^. This exposes ice-rich permafrost protected beneath previously undisturbed slopes, or underlying the floors of previously stabilised RTS and triggers new RTS. Fluvial, coastal and lacustrine erosion remain important over the long-term, acting as pre-conditioners to RTS initiation by de-buttressing slopes through lateral erosion, vertical incision or thaw settlement. The major initiator of RTS on Banks Island, however, is a deepening thaw layer^23^ caused by particularly warm summers such as 1998^38^. A progressive rise in mean summer air temperature due to climate change, punctuated by positive deviations from that moving average, is therefore expected to trigger new RTS (Fig. 2d).

### Impacts on water bodies

**Fig. 7:**
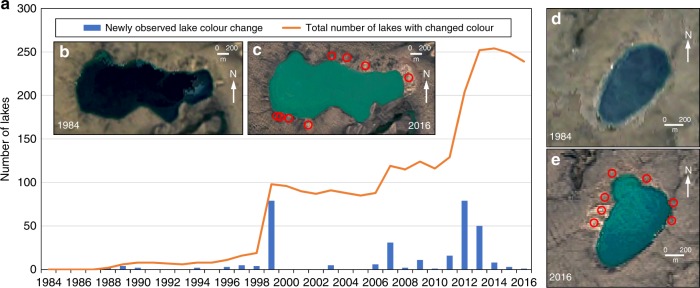
Lake colour change due to retrogressive thaw slump activity. **a** Annually resolved time series of numbers of lakes visually changing colour (from dark blue to turquoise or beige) observed for Banks Island (1984–2016) using Timelapse. **b**, **c** Individual lake colour change from 1984 to 2016 due to retrogressive thaw slumps becoming active around the shoreline (73.08°N 117.67°W). **d**, **e** Example of the development of a new bay in a lake due to retrogressive thaw slump activity (71.65°N 121.91°W). More than 200 m of shoreline recession occurred from 2000–2016. Red circles in (**c** and **e**) are locations of retrogressive thaw slumps which became active during the Timelapse period

Three lakes (each <1.3 ha in area) were infilled completely by sediment from adjacent RTS. A further 285 lakes were impacted sufficiently to change colour in the satellite imagery, from dark blue to turquoise or beige (Fig. 7a–c), presumably as a result of increases in the concentration of sediment suspended in the water column. All but seven were located in the Jesse Moraine where fines contents (silt and clay) in the till average 59%^39^, representing significant quantities for potential suspension. More than 95% of these lakes had RTS on their shores or on slopes leading into the lake while 4% were impacted by RTS that were active up-basin. The colour changes affected lakes of all sizes, including the largest on the island (63 km^2^), which developed 83 active RTS on its shorelines (Supplementary Video 1).

The temporal pattern of lake colour change followed that of RTS initiation: the two summers with the highest number of observed changes were 1999 and 2012 (79 in each year). The number of lakes impacted simultaneously reached a peak of 254 in 2014 and the probability of changes persisting for more than 15 years was on average 63%, compared to the probability of RTS remaining active for the same duration of 79%. These results, and the observation that some lakes changed colour intermittently despite an adjacent RTS being continuously active, demonstrate that visible effects can vary through time, likely depending on the strength of the hydrological connections between the retrogressing headscarp where water and sediment are generated and the lacustrine system at the RTS outlet^24,40^.

The impacts of the RTS on aquatic ecosystems in the region require additional investigation. Four fish species are present in lakes that were sampled in southwest Banks Island^41^ but our dataset shows that none of these lakes has been affected by RTS activity since 1984. The complexity of ecosystems in the lakes actually affected by RTS is unknown, as are the impacts of additional sediment loading and changes in solutes^24,40^. However, the new RTS dataset could be used to set up a well-constrained sampling scheme to examine the intensity and timing of impacts.

The Timelapse images suggest a unique RTS triggering mechanism at six lakes ranging in size from 50–900 ha. RTS developed contemporaneously with rapid recession of the shoreline (5–22 m/year), possibly due to wave action and thermal abrasion^3^. Timelapse revealed the formation of entirely new bays in the shorelines of these lakes (Fig. 7d and e; Supplementary Video 7).

River valleys were also impacted by sediment generated by RTS^22,23,28^. Catchments in the eastern part of the island, in particular, were subject to extensive valley floor sedimentation from 1999 onwards and sediment was transported as far as the ocean^23^ (Supplementary Videos 2, 8 and 9). The quantity of organic carbon exported remains unknown at present.

### Empirically modelled RTS initiation rates during the 20^th^ and 21^st^ Centuries

**Fig. 8:**
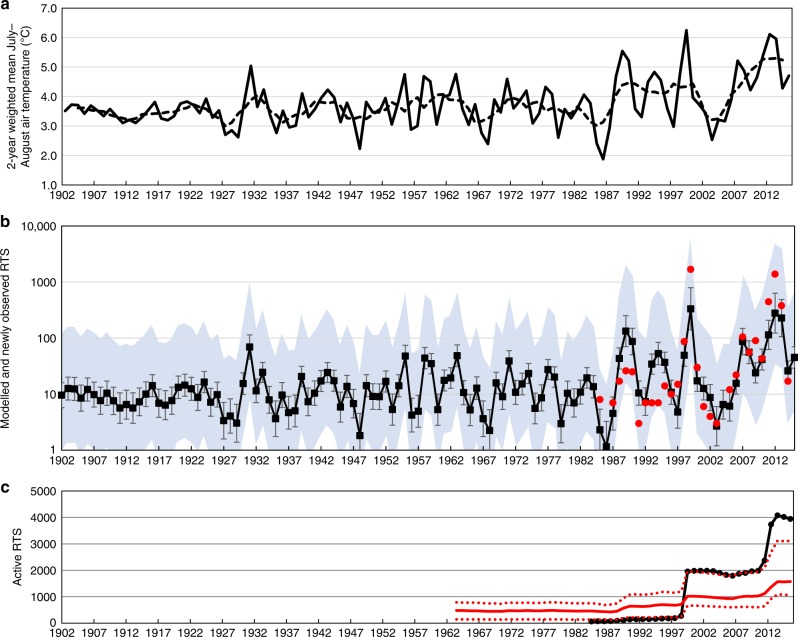
Summer air temperatures and hindcast retrogressive thaw slump activity. **a** 2-year weighted mean July–August air temperature (derived from the Hadley CRU dataset^52,53^) for Banks Island (1902–2015); dashed line is a 5-year centred running mean. Note: lower variance for 1901–1925 is due to constant values appearing in the Hadley CRU dataset for the northern part of the island for this period. **b** Hindcast retrogressive thaw slump initiation for 1902–2015 using the climate data in **a** and the relation in Fig. 2d. Error bars are 95% confidence intervals, shaded area represents prediction intervals, and red points are observed values. **c** Total number of retrogressive thaw slumps active on Banks Island (1964–2015). Solid red line was hindcast using initiation numbers in **b** and longevity relation in Fig. 3a. Dashed red lines are predictions made using upper 95% confidence interval for initiation and for longevity (Fig. 3a) and with lower 95% confidence interval for initiation and longevity. Black line is observed total number of active retrogressive thaw slumps (1984–2015)

**Fig. 9:**
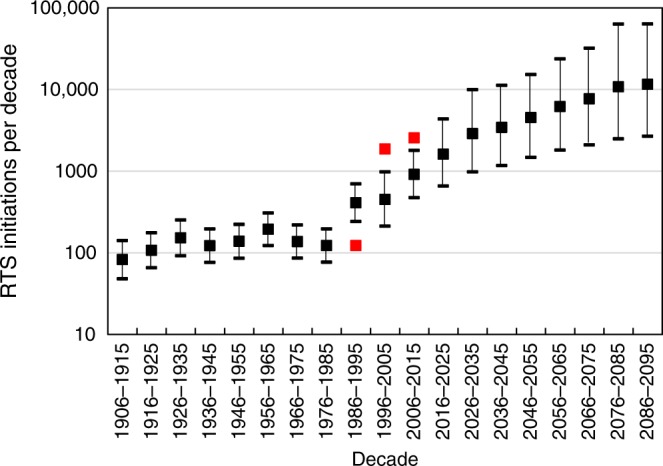
Modelled decadal retrogressive thaw slump initiation rates for Banks Island (1906–2015) and multimodel mean predictions under RCP4.5 (2016–2095). Red squares are observations based on the Timelapse data. Error bars for hindcast modelling generated using the Hadley CRU dataset^52,53^ are 95% confidence intervals. Values in the first two decades are affected by constant values appearing in the dataset for the northern part of the island. Future modelled mean and error bars are averages of 100 sets of predictions made with the best-fit relation in Fig. 2d and with 95% confidence intervals

Modelled RTS initiation rates in the 20^th^ century prior to 1985 range from 83 decade^−1^ (95% confidence: 48–142) for 1906–1915 to 195 decade^−1^ (95% confidence: 123–309) for 1956–1965 (Fig. 8b). These rates reflect relatively low mean summer air temperatures for Banks Island and the absence of extremely warm July–August temperatures for most of the century (Fig. 8a). The modelled rate is highest for 2006–2015 (915 decade^−1^; 95% confidence 476–1802), but this is conservative relative to the observed rate for the same period of 2542 decade^−1^ (Fig. 9).

In contrast, the modelled total number of active RTS, calculated from initiation and longevity rates, is greater than that observed at the start of the Timelapse period (Fig. 8c). This suggests that the average longevity of RTS earlier in the century may have been less than during 1985–2015. Because RTS must reactivate each summer, cold summers in the years following initiation could reduce longevity, and conversely, warm or wet^14^ summers could enhance it. This is supported by visual observations during analysis of the Timelapse dataset which indicated enhanced areal RTS growth during the warmest summers.

Modelled RTS initiation rates for the 21^st^ Century, represented as the decadal means of 100 model runs, increase from 1616 (95% confidence 660–4370) in 2016–2025, to 11,580 (95% confidence 2693–63,821) in 2086–2095 (Fig. 9). These predictions indicate, therefore, that a further order of magnitude increase in RTS numbers could occur by the end of the 21^st^ century. This forecast requires examination because several factors could intervene to affect the empirical relationships on which it is based.

First, it is possible that the relationship in Fig. 2d could be affected downwards by geomorphic change associated with greater RTS activity. Sedimentation in river valleys^23^ might raise the local base level and create negative feedback to prevent or slow down renewed initiation. Our observations, however, are that sedimentation of sufficient magnitude to be visible in Timelapse imagery, generally occurs well downstream of RTS sources where rivers debouch from narrow valleys and water velocities likely slow (Supplementary Video 9). Few RTS are present in these lower reaches of rivers. Furthermore, RTS initiated at coasts, on lakes or on slopes are not subject to this type of base level change. Consequently, while we cannot rule out this source of negative feedback, we believe that its impact on future activity will be minor.

Second, warm summers, occurring at a sub-decadal frequency in the future, might individually have less of an impact on RTS initiation. This hypothesis is based on the idea that deep thaw in antecedent years would reduce ice contents in the transient layer^42^, diminishing the likelihood that repeated high rates of late-summer thaw would generate elevated porewater pressures. This possibility is supported by the number of new RTS appearing in the southern part of Banks Island in 1999 and 2012 (see Fig. 6). Nearly 1000 RTS were first observed in the earlier year when the weighted July–August air temperature index for Sachs Harbour was 8.1 °C, while half that number (487) appeared in 2012, even though the index was higher (9.1 °C). This suggests that the response to the 1 °C increase may have been dampened by the deep thaw 13 years earlier. On the other hand, hundreds of RTS were initiated in all parts of the island as a result of three successive warm summers (2010–2012) (Fig. 6b) demonstrating that even if dampened, numbers remained high.

A third question relates to the availability of terrain susceptible to RTS formation if rates climb as predicted. As mentioned above, active RTS still occupy only a small percentage of the landscape: about 0.1% of the entire island or 0.5% of the 13,000 km^2^ of terrain in which at least 1 RTS per 25 km^2^ was active (see Fig. 5). Therefore, an order of magnitude increase in RTS numbers can be physically accommodated. Moreover, numerous valley side segments, lakeshores and coasts exhibit the scars of stabilised RTS (see Fig. 1b), where no activity was detected during the Timelapse period. Since polycyclicity is common (Supplementary Videos 3 and 6), RTS numbers could increase within the boundaries of these previously impacted areas, especially given the extensive ground ice present in the Wisconsinan age moraine belts on Banks Island^15,23^. Finally, RTS were observed in entirely new locations across Banks Island during the high initiation years of 2011–2013 which suggests that vulnerable areas may exist at the landscape scale outside the current concentrations. RTS could be initiated in these areas as the trend towards warmer summers results in the thaw of high ice content material buried at greater depths.

A final point relates to RTS longevity. Observations and our hindcasting (see above) suggest that RTS may remain active longer if temperatures increase. The impact of such a change in longevity would be to increase the number of active RTS in the landscape at any given time, independent of the number of initiations.

We conclude that the empirical relationships used for our modelling may be affected by changes in response as mean summer temperatures warm, but that sizable positive deviations from the mean will continue to initiate large numbers of RTS within and potentially outside the current areas of activity. These changes will develop more quickly than predicted if climate forcing exceeds the relatively moderate RCP4.5 scenario we used for our modelling.

## Discussion

We visually observed a 60-fold increase in RTS numbers on Banks Island from 63 in 1984 to a maximum of 4077 in 2013. The vast majority of these new thermokarst features were first observed in the Timelapse dataset in the years immediately following the four warm summers of 1998, 2010, 2011 and 2012. Most RTS remain active for several decades so even a single warm summer has a long-term effect on the landscape. The higher frequency of such events in the past 20 years has created cumulative impacts that have profoundly affected slopes, rivers and lakes on Banks Island. Our modelling indicates that current activity levels far exceed those of most of the 20^th^ Century and that a further substantial increase in RTS formation and activity should be expected over the next 80 years as the climate warms.

This is the first study in which RTS numbers have been compiled with an annual resolution over a very large area, an analysis made possible by the prior assembly of satellite imagery in the Timelapse dataset. The newly derived RTS dataset for Banks Island will be useful for multiple purposes, such as validation of automated remote sensing methods^35,43^, for landscape susceptibility modelling^11^, and to managers of the Aulavik National Park where 300 RTS were active in 2015. Visualisation using Timelapse also means that videos of selected sites can be used as educational tools (Supplementary Videos 1–9).

The areal expansion of significant RTS activity into northern Banks Island from 2007 onwards is a direct response to regional warming and suggests that areas of ice-rich permafrost elsewhere in the Arctic, which may be relatively unaffected by thermokarst at present due to low summer air temperatures, could become impacted as the climate warms over the 21^st^ century. A similar areal expansion has occurred in more southerly regions over the past several decades^14,15,29^. The order of magnitude increase that we observed and the further increase projected will make these areas of the Arctic some of the most dynamic on the planet in terms of landscape change^1,5,28^, with impacts on ecosystems that remain largely unknown.

## Methods

### Study area

We tracked RTS activity across Banks Island (area 70,000 km^2^), the westernmost and fourth largest island in the Canadian Arctic Archipelago^39^. The terrain across the island varies from a dissected upland plateau of Devonian age bedrock in the north with elevations exceeding 350 m above sea level (asl), to the hilly ice-rich Late Wisconsinan Sand Hills Moraine in the southwest (produced by a readvance of a lobe in the Amundsen Gulf) and the similarly hummocky Jesse Moraine belt in the east^39,44^. Rolling lowlands dominate the terrain in the western part of the island while a bedrock highland exceeding 650 m asl is present at its southern tip.

The climate of Banks Island is cold and dry. There are three official climate stations (Fig. 6b): Sachs Harbour (1956-present), on the coast in the extreme southwest (71.99°N, 125.24°W); Thomsen River (1997–present) in the north-central part of the island (73.23°N, 119.54°W); and Nangmagvik Lake (1996–present) near the coast in the far north (74.14°N, 119.94°W). The mean annual air temperature (1981–2010) at Sachs Harbour is −12.8 °C and annual precipitation totals 152 mm, 65% of which falls as snow^45^. Three vegetation types cover more than 90% of the island: the Jesse moraine belt mainly supports prostrate dwarf-shrub herb tundra; graminoid prostrate dwarf-shrub forb tundra covers the central rolling hills and valleys; cryptogam-herb barrens are present on upland areas in the north^46^. Limited low elevation areas along the west and south coasts are classified as sedge/grass moss wetlands and small areas in the extreme south of the island exhibit either nontussock, sedge, dwarf shrub, moss tundra or low-shrub tundra^46^.

Banks Island lies entirely within the zone of continuous permafrost^47^ with ground ice contents varying from high to medium-to-high. Ground temperatures at Thomsen River in north-central Banks Island average about −12 °C (3 m depth; 2010–2015)^48^ and permafrost thickness on the island can exceed 500 m^47^. Both the Jesse Moraine and the Sand Hills Moraine contain ground ice that has been interpreted as glacial in origin^44,49^ and these areas have long been known as subject to thermokarst processes^33,50,51^.

### Use of Timelapse

We used the Google Earth Engine Timelapse dataset^31^ to visually assess terrain change across Banks Island. Timelapse is a series of annual cloud-free satellite images of the entire globe dating from 1984 to 2016 arranged as a zoomable timelapse video. The maximum resolution depends on the satellite platform that acquired the image. At the resolution employed for this study, the platforms were Landsat 4, 5, 7 and 8, and Sentinel -2A^31^ generally with pixel sizes of 30 × 30 m or smaller (Supplementary Table 2). It is possible that some of the imagery for the years 1984–1993 is from Landsat 4 with a resolution of 60 × 60 m, but visual observation suggested otherwise. Imagery covering Banks Island can generally be zoomed on-screen to a scale of ~1:15,000.

Active RTS were detected visually while viewing the image sequence on the fast setting which causes progressive change to appear as feature motion (Supplementary Videos 1–9). Consequently, RTS as small as 0.2 ha were identifiable (Supplementary Video 10). Frame by frame inspection was then used to determine the dates of activity with an annual resolution. At numerous sites, RTS were polycyclic, with two or even three generations of headwalls transgressing simultaneously (Supplementary Video 3).

Our method is time-consuming compared to automated techniques to detect change, such as optical reflectance trend analyses^32,35^. However, it has the advantage that the cause of observed change is usually identifiable. It therefore reduces confusion with image changes relating to factors such as the formation of active layer detachments or other landslides. Compared to traditional assessments with images separated by years or decades, it has the advantage of allowing a determination of actual activity, rather than inferring activity from headscarp appearance or lack of vegetation^28^.

The major advantage of the Timelapse dataset is that it is already assembled and can be accessed without specialised software or equipment^31^, meaning that large areas can easily be examined. The inherent drawback is that the source and date of acquisition of any given image (or part of an image) is opaque to the user. During assembly, pixels are selected as being representative of a given year so that an early season land cover change might be included while a late-season change might be rejected as an outlier. In some years, no appropriate data may be available for a given site leading to interpolation between years. Given our visual analysis, the main impact of these pre-treatments is to create a degree of uncertainty regarding the timing of RTS initiation and activity cessation.

The initial survey of the island was carried out in duplicate by a class of senior undergraduate students at the University of Ottawa, with each student allocated an east–west transect of about 5000 km^2^. The results were compiled and trained student research assistants reviewed and added to the initial dataset of >1000 RTS, quadrupling the total numbers. The dataset for the entire island was examined by the first author, and a second review was undertaken for the areas with recorded RTS. In total, therefore, all areas with RTS were examined five times. The approach taken was to minimize false positives so that if there was doubt about the origin of an apparent change, it was not retained in the dataset. The mapped changes are therefore most likely to be an underestimate of the entire population, especially of (1) RTS smaller than 0.2 ha, (2) those which were active for less than three years, and (3) those that were initiated close to the end of the record. For this reason, even though Timelapse includes 2016 images, initiation rates are presented to 2014 and total active RTS numbers to 2015.

Once identified, each active RTS was given a unique number and classified according to its point of initiation (Supplementary Data 1). The four RTS types recognised were initiated next to a river channel (R), at the coast (C), on a lakeshore (L), or on a slope (S) away from a river, lake or the coast (Fig. 1b). Slope RTS included those initiated in the floor of an older previously stabilised or still-active feature.

The year that each active RTS was first observed was recorded. This date incorporates uncertainty of 0 to +1 year because initiation could have occurred prior to the acquisition of a specific portion of the Timelapse image in a given summer or after that image was acquired. Furthermore, even if the former was the case, the RTS might not have become large enough to be visible until the following year. The year of apparent stabilisation, recognised by cessation of headwall retrogression, was also recorded. The duration of activity was calculated as inclusive of the recorded start and end years.

RTS were recognised as individual features if their points of initiation were non-contiguous. Two or more RTS which started on the same river reach, for example, but eventually conjoined laterally (or in a few cases as their headwalls from adjacent valleys met on an interfluve) were counted as two features in the dataset.

RTS locations, together with the ancillary information described above, were recorded as points on Google Earth (Fig. 1b). Most of the Google Earth satellite imagery for Banks Island dates to 2016, although earlier images can also be viewed. Parts of eastern Banks Island are covered by high-resolution Quickbird imagery dating from 2004 and 2006. Temporal and spatial calculations and statistical analyses were carried out in Excel 16.11.1 for Mac after export of the dataset from Google Earth, and maps were generated in ESRI ArcGIS 10.6.

A sample of RTS was digitized on available Google Earth imagery to establish an average growth curve in order to calculate the total area affected by RTS. Fifty RTS in each of the seven highest initiation years were selected in a spatially stratified sample. In addition, the entire population of RTS initiated from 1987–1990 was included. The final sample was reduced to *n* = 341 as some RTS were initiated after the available image so they could not be measured and in a few cases, the resolution of the Google Earth imagery was too poor to allow confidence in the RTS outlining procedure. The area of an RTS that conjoined during its development with another numbered feature was calculated as a weighted average based on the combined area and the number of years of activity of each of the component RTS. Conjoined RTS were excluded from the sample where the initiation dates differed by more than five years. Because some RTS in the sample stabilised prior to the image date, their ages varied more than their initiation dates (Fig. 3b).

The average areal growth rate was applied to each individual RTS to estimate the total area of Banks Island affected over the Timelapse period. The area subject to RTS at the start of the record was established by examining each active RTS in Timelapse for 1984 and outlining its margins for that year on Google Earth, assuming that it initiated at the local base level. These RTS were given estimated ages based on their areas and then allowed to expand along the RTS growth line until their observed date of stabilisation (if any).

The initial surveys showed that numerous lakes with RTS on their shorelines changed colour from dark blue to turquoise or in a few cases to beige, over the period of record and some expanded dramatically. These changes were recorded (Supplementary Data 2), as were the location and timing of substantial sedimentation in catchments subject to intense RTS activity.

### Validation of the Timelapse method

Ten RTS were recorded as being active in part of the Sand Hills moraine during fieldwork in 1984, mostly along the coast^33^. All were identifiable in the Timelapse dataset. We are not aware of any other published field observations for Banks Island that are available to validate our historical results.

Our results could be compared, however, to another remote sensing survey^23^ carried out using a 2015 high-resolution WorldView 2 satellite image of the 230 km^2^ Johnson Point watershed in eastern Banks Island. Our database showed 134 RTS as being active in the basin in 2015, more than 90% of which formed part of the 153 active RTS identified in the previous study^23^. Where there was disagreement between the two surveys, we re-examined the Timelapse images closely. Our assessment is that our survey omitted 12 RTS (8% of the total), mostly small features (average size 0.36 ha; maximum 0.88 ha) while two other small identified features (2%) may not have been RTS (Table 1). We also judged that we correctly identified 10 RTS which had been missed in the previous work^23^ and that 19 previously identified disturbances were not active RTS in 2015. This analysis indicates that our island-wide RTS dataset should have fewer than 5% false positives but that up to 10% of active RTS, principally small features, may not have been detected. This size bias preferentially affects the youngest features, so post-2012 activity, or earlier RTS that were active only for a short time, are most subject to underestimation. We conclude that our dataset is at least as complete as earlier surveys carried out over limited areas, and that while it may underestimate the increase in activity during 1984–2015, it should not overestimate the change.

**Table 1:** Error matrix for identification of active retrogressive thaw slumps in 2015 for the Johnson Point watershed

	RTS identified as active	RTS mis-identified as active	Active RTS not identified
This study	134 (93%)	2 (1%)	12 (8%)
Rudy et al.^23^	153 (106%)	19 (13%)	10 (7%)

Percentages are calculated in relation to the estimated total number of RTS active in the catchment in 2015 (144)

### Climate data

We assessed the link between summer climate and RTS initiation for 1984–2015 using the Hadley CRU 4.0 surface air temperature dataset^52,53^ with values extracted and averaged for all the grid cells covering Banks Island (71–75°N and 117–126°W). For hindcasting during the 20^th^ Century, we used the same dataset from 1901 onwards. However, there was reduced variance in the dataset for the period 1901–1925 because climatological averages were used for the northern part of the island in these years due to an absence of any proximal climate station. In all cases, we calculated a July–August air temperature index weighted as 66.7% of the previous year and 33.3% of the current year. Other indices were tried, taking into account June or September monthly values or with differing inter-annual weightings, but the chosen index provided the best fit with RTS initiation during the Timelapse period.

We used individual air temperature records from the three climate stations on Banks Island to examine spatial changes in RTS response (Fig. 6). We used a homogenized dataset^54,55^ for Sachs Harbour while data for Thomsen River and Nangmagvik Lake were extracted directly from station records^56^. All three stations had missing monthly data, but not in those summers when large numbers of RTS were first observed. Regression with the best available predictor station was used to infill individual months. Infilled air temperatures for Sachs Harbour were based on the Holman/Ulukhatok station located 300 km to the southeast on Victoria Island (total of eight months; July: *n* = 32, SE = 1.2 °C, *r*^2^ = 0.75; August: *n* = 31, SE = 0.7 °C, *r*^2^ = 0.87). Infilling for Thomsen River was based on Sachs Harbour, 230 km to the south (total of two months; July: *n* = 17, SE = 0.7 °C, *r*^2^ = 0.88; August: *n* = 17, SE = 0.9 °C, *r*^2^ = 0.75). For Nangmagvik Lake, infilling used records from Thomsen River, located 85 km to the south (total of four months; July: *n* = 16, *r*^2^ = 0.90, SE = 0.7 °C; August: *n* = 16, *r*^2^ = 0.90, SE = 0.5 °C) or in their absence, records from Mould Bay located 230 km to the north on Prince Patrick Island (total of two months; July: *n* = 16, SE = 1.6 °C, *r*^2^ = 0.44; August: *n* = 17, SE = 1.0 °C, r^2^ = 0.77).

We investigated potential links between RTS initiation and summer rainfall which has been shown to be important in some more southerly regions^14,16^. We first compared July–August precipitation recorded at the three stations on Banks Island to examine whether there was spatial homogeneity. The coefficient of determination between Sachs Harbour and Thomsen River was 0.24 (*n* = 11) while it was 0.04 (*n* = 14) between Sachs Harbour and Nangmagvik Lake. These low correlations demonstrate the difficulty of interpolating rainfall across distances of 200–300 km. Furthermore, reconstructions of precipitation in the Arctic Archipelago are also subject to considerable uncertainty^57^. Given the limited record for the two northerly stations, we decided to compare Sachs Harbour alone to the logarithm of RTS initiation employing a July–August precipitation index with the same weighting used for temperature (66.7% from the previous year and 33.3% from the current year). There was no significant relationship (*r*^2^ = 0.025; *n* = 27). We also undertook a multiple regression analysis with both the July–August weighted air temperature index and the precipitation index as independent variables but the latter proved statistically non-significant. The absence of statistical significance could be because the Sachs Harbour data are insufficiently representative of the major area of RTS on the east and north coasts, as suggested by the low correlations, or because a strong causal relationship is lacking. While we cannot entirely rule out the possibility that precipitation plays a role in RTS initiation on Banks Island, therefore, such a relationship was not supported by the data examined.

For future modelling we used July and August monthly air temperatures generated from a multimodel mean (42 models; Supplementary Table 3) based on RCP4.5, selected as a moderate warming scenario. We calculated 2-year weighted mean July–August temperatures for the period 2016–2100 and smoothed the resultant values using an 11-year running mean. We then generated 100 potential records by randomly adding or subtracting deviations based on a normal distribution from the running mean, with the standard deviation equal to that of the Hadley CRU data for 1986–2015 (1.1 °C). Each record series was used with the relations in Fig. 2d (best-fit, +95% confidence interval, and −95% confidence interval) to produce a time series of RTS initiations. Each of the three sets of initiations was then averaged to produce decadal predictions (Fig. 9).

Our predictions are potentially statistically conservative as the three highest years of record in Fig. 2d all fall above the log-linear line. In fact, a second-order polynomial provides a closer fit to the highest points and yields a higher *r*^2^ value (0.72 vs. 0.64) but given the logarithmic dependent variable, this would yield RTS initiations for slightly higher July–August temperatures that are several orders of magnitude outside the range of the record to date. For this reason, we concluded that the log-linear empirical relationship shown in Fig. 2d was more appropriate for both the hindcasting and future modelling that we undertook.

## Supplementary information

Supplementary Information

Description of Additional Supplementary Files

Supplementary Data 1

Supplementary Data 2

Supplementary Video 1

Supplementary Video 2

Supplementary Video 3

Supplementary Video 4

Supplementary Video 5

Supplementary Video 6

Supplementary Video 7

Supplementary Video 8

Supplementary Video 9

Supplementary Video 10

## Data Availability

The RTS dataset is given in Supplementary Data 1 and the lake colour change dataset is in Supplementary Data 2.
