# Supplementary material for: Extremes of summer climate trigger thousands of thermokarst landslides in a High Arctic environment
Source: Nat Commun. 2019 Apr 2;10:1329. doi: 10.1038/s41467-019-09314-7 (PMC6445831; doi:10.1038/s41467-019-09314-7)
Supplement: Supplementary file 1 — Supplementary Information [file 41467_2019_9314_MOESM1_ESM.pdf]

Supplementary Information for

Extremes of summer climate trigger thousands of thermokarst landslides in a High Arctic environment

Antoni G. Lewkowicz and Robert G. Way

Supplementary Table 1: List of Timelapse videos of selected examples of retrogressive thaw slump development on Banks Island (1984-2016).

**Video**	**Latitude (°N)**	**Longitude (°W)**	**Retrogressive thaw slump (RTS) characteristics**
1	73.08	118.10	Largest contiguous area affected by RTS on Banks Island.
2	71.91	120.64	Exceptionally high density of RTS causing valley sedimentation.
3	71.55	122.01	Polycyclic RTS initiated on rivers.
4	73.08	117.66	RTS initiated on a lakeshore.
5	71.65	123.88	RTS initiated on slopes.
6	71.72	124.12	Polycyclic RTS initiated at the coast.
7	71.65	121.91	RTS triggered by lake expansion and bay formation.
8	72.82	118.50	Slope RTS contributing sediment into the fluvial system.
9	71.69	120.50	Export of sediment produced by inland RTS activity starting in 1999 to Prince of Wales Strait.
10	73.50	115.54	RTS on rivers in eastern Banks Island showing features varying in area from 0.2-1.5 ha (black numbers). Arrows point to location of RTS headscarps in 2016 and are oriented parallel to the long axes of RTS. Arrow colour denotes year of initiation: black – 2009, yellow – 2011 and red – 2012. RTS areas in 2016 obtained by outlining features on Google Earth image.

Note: videos show changes over the Timelapse period repeated three times. Coordinates given are the centre of the area shown in each video.

Supplementary Table 2: Satellite platforms and sensors potentially used in the Timelapse dataset[31]

Year	Satellite and sensor(s)	Multispectral spatial resolution(s)	Panchromatic band(s)
1984	Landsat 4 MSS / Landsat 5 TM	60 m / 30 m	NA
1985	Landsat 4 MSS / Landsat 5 TM	60 m / 30 m	NA
1986	Landsat 4 MSS / Landsat 5 TM	60 m / 30 m	NA
1987	Landsat 4 MSS / Landsat 5 TM	60 m / 30 m	NA
1988	Landsat 4 MSS / Landsat 5 TM	60 m / 30 m	NA
1989	Landsat 4 MSS / Landsat 5 TM	60 m / 30 m	NA
1990	Landsat 4 MSS / Landsat 5 TM	60 m / 30 m	NA
1991	Landsat 4 MSS / Landsat 5 TM	60 m / 30 m	NA
1992	Landsat 4 MSS / Landsat 5 TM	60 m / 30 m	NA
1993	Landsat 4 MSS / Landsat 5 TM	60 m / 30 m	NA
1994	Landsat 5 TM	30 m	NA
1995	Landsat 5 TM	30 m	NA
1996	Landsat 5 TM	30 m	NA
1997	Landsat 5 TM	30 m	NA
1998	Landsat 5 TM	30 m	NA
1999	Landsat 5 TM / Landsat 7 ETM+	30 m / 30 m	NA / 15 m
2000	Landsat 5 TM / Landsat 7 ETM+	30 m / 30 m	NA / 15 m
2001	Landsat 5 TM / Landsat 7 ETM+	30 m / 30 m	NA / 15 m
2002	Landsat 5 TM / Landsat 7 ETM+	30 m / 30 m	NA / 15 m
2003	Landsat 5 TM / Landsat 7 ETM+	30 m / 30 m	NA / 15 m
2004	Landsat 5 TM / Landsat 7 ETM+	30 m / 30 m	NA / 15 m
2005	Landsat 5 TM / Landsat 7 ETM+	30 m / 30 m	NA / 15 m
2006	Landsat 5 TM / Landsat 7 ETM+	30 m / 30 m	NA / 15 m
2007	Landsat 5 TM / Landsat 7 ETM+	30 m / 30 m	NA / 15 m
2008	Landsat 5 TM / Landsat 7 ETM+	30 m / 30 m	NA / 15 m
2009	Landsat 5 TM / Landsat 7 ETM+	30 m / 30 m	NA / 15 m
2010	Landsat 5 TM / Landsat 7 ETM+	30 m / 30 m	NA / 15 m
2011	Landsat 5 TM / Landsat 7 ETM+	30 m / 30 m	NA / 15 m
2012	Landsat 5 TM / Landsat 7 ETM+	30 m / 30 m	NA / 15 m
2013	Landsat 7 ETM+ / Landsat 8 OLI	30 m / 30 m	15 m / 15 m
2014	Landsat 8 OLI	30 m	15 m
2015	Landsat 8 OLI / Sentinel 2A MSI	30 m / 10 m	15 m / NA
2016	Landsat 8 OLI / Sentinel 2A MSI	30 m / 10 m	15 m / NA

Supplementary Table 3: Models used to generate predictions of future summer air temperatures for Banks Island under RCP4.5

ACCESS1-0	FGOALS-g2	HadGEM2-CC
ACCESS1-3	FIO-ESM	HadGEM2-ES
bcc-csm1-1	GFDL-CM3	inmcm4
bcc-csm1-1-m	GFDL-ESM2G	IPSL-CM5A-LR
BNU-ESM	GFDL-ESM2M	IPSL-CM5A-MR
CanESM2	GISS-E2-H	IPSL-CM5B-LR
CCSM4	GISS-E2-H	MIROC5
CESM1-BGC	GISS-E2-H	MIROC-ESM
CESM1-CAM5	GISS-E2-H-CC	MIROC-ESM-CHEM
CMCC-CM	GISS-E2-R	MPI-ESM-LR
CMCC-CMS	GISS-E2-R	MPI-ESM-MR
CNRM-CM5	GISS-E2-R	MRI-CGCM3
CSIRO-Mk3-6-0	GISS-E2-R-CC	NorESM1-M
EC-EARTH	HadGEM2-AO	NorESM1-ME
