# Supplementary material for: Extremes of summer climate trigger thousands of thermokarst landslides in a High Arctic environment
Source: Nat Commun. 2019 Apr 2;10:1329. doi: 10.1038/s41467-019-09314-7 (PMC6445831; doi:10.1038/s41467-019-09314-7)
Supplement: Supplementary file 2 — Description of Additional Supplementary Files [file 41467_2019_9314_MOESM2_ESM.pdf]

Description of Additional Supplementary Files

File Name: Supplementary Data 1
Description: Retrogressive thaw slump activity dataset (1984-2015)

File Name: Supplementary Data 2
Description: Lake colour change dataset (1984-2015)

File Name: Supplementary Video 1
Description: Google Earth Engine Timelapse video of the largest contiguous area affected by retrogressive thaw slumping on Banks Island. The disturbed area is approximately 2.5 km wide and extends 0.5 km in from the lakeshore where most of the polycyclic slumps are triggered following the warm summer of 1998. Note: the video repeats the temporal sequence from 1984-2016 three times. Location: 73.08°N, 118.10°W.

File Name: Supplementary Video 2.
Description: Google Earth Engine Timelapse video of very high concentration of retrogressive thaw slumps triggered along river valleys following the warm summer of 1998 and resulting in accumulation of sediment downstream (top right). Note: the video repeats the temporal sequence from 1984-2016 three times. Coordinates: 71.91°N, 120.64°W.

File Name: Supplementary Video 3.
Description: Google Earth Engine Timelapse video of polycyclic retrogressive thaw slumps initiated along rivers. Three slumps are active at the start of the period in 1984 (central part of window) while new slumps become visible in their floors in 1999 and remain active until the end of the record. Note: the video repeats the temporal sequence from 1984-2016 three times. Coordinates: 71.55°N, 122.01°W.

File Name: Supplementary Video 4.
Description: Google Earth Engine Timelapse video of retrogressive thaw slumps initiated along a lakeshore (see Fig. 7b and c). There are no active slumps around the lake before 1999 when three become visible and the lake colour changes from dark blue to turquoise. Five more slumps are triggered in 2012 and 2013. Note: the video repeats the temporal sequence from 1984-2016 three times. Coordinates: 73.08°N, 117.66°W.

File Name: Supplementary Video 5
Description: Google Earth Engine Timelapse video of retrogressive thaw slumps initiated on slopes. There are no active slumps before 1999 when three become visible in the central area with a fourth towards the top of the window. Their activity causes significant sedimentation downslope. An additional thaw slump is initiated in the floor of the largest feature in 2009 and another on the slope in 2013. Note: the video repeats the temporal sequence from 1984-2016 three times. Coordinates: 71.65°N, 123.88°W.

File Name: Supplementary Video 6.
Description: Google Earth Engine Timelapse video of polycyclic retrogressive thaw slumps initiated at the coast. More than 20 slumps were active in this part of the Sand Hills moraine

during the Timelapse period and almost all were polycyclic, forming in the floors of older stabilized or still-active slumps. Note: the video repeats the temporal sequence from 1984-2016 three times. Coordinates: 71.72°N, 124.12°W.

File Name: Suppleme
ntary Video 7.
Description: Google Earth Engine Timelapse video of retrogressive thaw slumps triggered by lake expansion and bay formation (see Fig. 7d and e). The lake colour change is coincident with thermokarst activity that starts in 1990. Note: the video repeats the temporal sequence from 1984-2016 three times. Coordinates: 71.65°N, 121.91°W.

File Name: Supplementary Video 8.
Description: Google Earth Engine Timelapse video of more than ten retrogressive thaw slumps initiated on slopes from 2011-2012 contributing sediment into the fluvial system. Note: the video repeats the temporal sequence from 1984-2016 three times. Coordinates: 72.82°N, 118.50°W.

File Name: Supplementary Video 9.
Description: Google Earth Engine Timelapse video showing fluvial export of sediment produced by inland retrogressive thaw slump activity from 1999 onwards to Prince of Wales Strait. Note: the video repeats the temporal sequence from 1984-2016 three times. Coordinates: 71.69°N, 120.50°W.

File Name: Supplementary Video 10.
Description: Google Earth Engine Timelapse video of retrogressive thaw slumps developed adjacent to rivers in eastern Banks Island showing features varying in area from 0.2-1.5 ha (black numbers). Arrows point to location of thaw slump headscarps in 2016 and are oriented parallel to the long axes. Arrow colour denotes year when individual retrogressive thaw slumps were first detected: black – 2009, yellow – 2011 and red – 2012. Thaw slump areas in 2016 were obtained by outlining features on the Google Earth image from that year. Note: the video repeats the temporal sequence from 1984-2016 three times. Coordinates: 73.50°N, 115.54°W.
